# Integration analysis using bioinformatics and experimental validation on cellular signalling for sex differences of hypertrophic cardiomyopathy

**DOI:** 10.1111/jcmm.70147

**Published:** 2024-11-13

**Authors:** Hongyu Kuang, Yanping Xu, Guangliang Liu, Yuhao Wu, Zhiyan Gong, Yuehui Yin

**Affiliations:** ^1^ Department of Cardiology The Second Affiliated Hospital of Chongqing Medical University Chongqing China; ^2^ Chongqing Key Laboratory of Cardiac Electrophysiology Chongqing China; ^3^ Department of Cardiothoracic Surgery Children's Hospital of Chongqing Medical University Chongqing China; ^4^ Department of Ultrasound the First Affiliated Hospital of Chongqing Medical University Chongqing China

**Keywords:** bioinformatic analysis, hypertrophic cardiomyopathy, prognostic biomarkers, sex differences

## Abstract

There is a paucity of research examining the molecular mechanisms underlying sex differences of clinical phenotypes and the prognosis in hypertrophic cardiomyopathy (HCM). The dataset GSE36961 was retrieved from Gene Expression Omnibus (GEO) database and comprehensive bioinformatics was employed to identify the core genes linked to sex differences in HCM patients. Additionally, gene set enrichment analysis (GSEA) was conducted to detect downstream signalling pathways. Furthermore, experimental validation was carried out using hearts from spontaneously hypertensive rats (SHRs). A comprehensive analysis revealed the identification of 208 differentially expressed genes (DEGs) in female patients with HCM with a notable downregulation of seven core genes. Notably, there were sex differences in the expression of ras dexamethasone‐induced protein 1 (RASD1) and myosin 6 (MYH6) in HCM. Gene ontology (GO) analysis and GSEA demonstrated an enrichment of autophagy‐related processes in disease progression in HCM females. Specifically, spearman's correlation analysis revealed a positive correlation between nicotinamide phosphoribosyl transferase (NAMPT) and RASD1 levels, particularly among female patients (*R* = 0.569, *p* < 0.001). Additionally, animal models confirmed that cardiac hypertrophy was more pronounced in SHR females compared to males. SHR females exhibited lower mRNA and protein expressions of RASD1 and NAMPT, which were associated with impaired autophagy. In this study, bioinformatics and validation using external data sets and animal models of left ventricular hypertrophy suggested that the RASD1/NAMPT axis is potentially a crucial mechanism underlying the elevated risk of cardiovascular disorders in HCM females, also pointing potentially prognostic biomarkers.

## 1 BACKGROUND

Hypertrophic cardiomyopathy (HCM) is the most prevalent inherited heart condition, affecting approximately 0.5% of adults.^1^ Characterized by left ventricular hypertrophy (LVH) without a known aetiology, it is primarily diagnosed through electrocardiography or cardiac MRI.^2^ While HCM exhibits clinical heterogeneity among individuals, including varying presentations, natural histories and the presence or absence of obstructive phenotypes, the underlying progression mechanism remains enigmatic.^3^, ^4^, ^5^

Recent evidence suggests that sex differences significantly contribute to the phenotypic heterogeneity among HCM patients. For instance, men are more prevalent among HCM cases, while women face a higher risk of cardiovascular outcomes and poorer survival rates.^6^, ^7^, ^8^, ^9^ Although a previous study failed to detect significant differences in cardiovascular mortality between HCM females and males, sex‐based differences were evident in cardiovascular outcomes, with a hazard ratio of 3.60 (95% CI: 2.00–6.49), indicating the crucial role of sex in HCM patients' cardiovascular risk.^10^ Furthermore, women with HCM exhibit poorer cardiovascular prognoses than men, attributed to a higher risk of cardiovascular events, including heart failure, atrial fibrillation and sudden cardiac death.^11^, ^12^

The aetiology of HCM is attributed to pathogenic variants in sarcomere genes encoding myofibroblasts, such as MYBPC3, MYH7, TNNT2, MYL2 and MYH6 Genetic and environmental factors contribute to the clinical phenotype and prognosis of HCM patients. Notably, sex is a crucial determinant of clinical characteristics and adverse outcomes in HCM, with women being more likely to have sarcomere variants (sarcomere‐positive), indicating a disease‐causing variant compared to men.^13^, ^14^ However, this does not fully explain the severity of symptoms observed in women with HCM.

As the genetic underpinnings of HCM, particularly those related to sex differences, remain enigmatic, this study aims to elucidate causative genes and molecular mechanisms explaining HCM of sex differences, as novel diagnostic biomarkers, thereby informing cardiac surveillance, enabling early detection and facilitating targeted treatments.^15^

## 2 METHODS

### 2.1 Gene expression data acquisition

The microarray gene expression profiles of cardiac tissues, derived from the GSE36961 dataset, were retrieved from the GEO database (https://www.ncbi.nlm.nih.gov) and annotated with GPL15389 as a Series Matrix File. A total of 106 cardiac samples were acquired from patients with HCM, including 54 males and 52 females, while 39 samples were obtained from the control group, comprising 19 males and 20 females.

### 2.2 Identification of differentially expressed genes

This study downloaded and reanalyzed microarray data from the publicly available GEO database, employing rigorous bioinformatics techniques. Differential expression analysis was conducted using an R software package (ggplot2 version 3.3.6), with a volcano plot utilized to identify genes exhibiting a log2 fold change (FC) ≥1 or log2 FC ≤ −1, combined with an adjusted *p* < 0.05. Given the clinical relevance of sex differences in HCM, our focus was on female patients, who are known to face a higher risk of cardiovascular complications, as reported in previous clinical trials.^10^ To identify DEGs in HCM females, we utilized the limma package, analysing samples from 52 female HCM patients and 20 female controls. Functional annotation of these genes was performed using the clusterProfiler package in R, with the Benjamini‐Hochberg (BH) method applied for *p*‐value adjustment. A *p*‐value cutoff of 0.05 was deemed statistically significant for both GO analysis and KEGG pathway enrichment analysis.

### 2.3 Construction of the LASSO model and SVM‐RFE model for identifying core genes

To identify the core genes associated with female‐related HCM, the LASSO logistic regression and SVM algorithm were employed. For the LASSO analysis, the ‘glmnet’ package in R software was utilized. To ensure more reliable and objective results, fivefold cross‐validation was applied to determine the optimal lambda (*λ*) value with minimal misclassification error. Additionally, SVM‐RFE was adopted to efficiently extract the best variables. These variables were then screened using the SVM module via the ‘e1071’ package in R software. Finally, the core gene subsets were identified by overlapping the genes using a Venn diagram generated from both the LASSO and SVM‐RFE models.

### 2.4 Gene set enrichment analysis (GSEA)

Based on the median expression level of the screened pivot genes,^16^, ^17^ the study cohort of 52 female patients with HCM was categorized into two distinct groups: the high‐expression group and the low‐expression group. To gain insights into the underlying biological processes (BPs), GSEA was conducted by comparing the expression profiles of genes within signalling pathways between the two groups. Leveraging the Molecular Signature Database (MSigDB) v7.0 and its hallmark gene sets, we obtained the necessary background gene set data for our analysis. We computed the consistency‐adjusted *p*‐value for each gene set, with a threshold of 0.05 considered statistically significant. Subsequently, the significantly enriched gene sets were ranked. Furthermore, GSEA was employed to explore the association between disease subtype and specific BPs.

### 2.5 Validation of core genes in men‐related HCM

To investigate the significance of seven identified core genes in human‐related HCM, we conducted a comparative analysis of their relative expression levels between HCM males (*n* = 54) and healthy males (*n* = 19) within the same dataset. Additionally, we compared the expression patterns of these core genes between tissues from HCM males and those from female‐related HCM patients to identify the most crucial genes and elucidate the underlying mechanisms that account for sex differences in their expression.

### 2.6 Spearman's correlation analysis

To investigate the impact of autophagy on sex differences in HCM, we employed Spearman's correlation analysis to identify correlations between the expressions of core genes and autophagy‐related genes obtained from the Human Autophagy Database.^18^ This comprehensive database offers a detailed listing of human genes and proteins that are either directly or indirectly involved in autophagy.

### 2.7 Animal models

Since the last century, the spontaneously hypertensive rats (SHRs) animal model has been considered as a solid model for studying pathological cardiac hypertrophy and heart failure.^19^, ^20^, ^21^ The SHRs aged about 6 months have exhibited the development of LVH in this study. Both SHR and WKY rats for this study were sourced from Vital River in Beijing, China. The Institutional Animal Experiments were granted approval by the Institutional Animal Care and Use Committees of Chongqing Medical University, China. All animals were given humane care, adhering to the guidelines established by the National Institutes of Health (USA). Given the study's focus on the potential mechanisms underlying sex differences in HCM, the experimental verification utilized WKY female rats (*n* = 6), WKY male rats (*n* = 6), SHR female rats (*n* = 6) and SHR male rats (*n* = 6).

### 2.8 Echocardiography

Using an ultrasonic diagnostic instrument (Esaote Mylab 90, Canada), echocardiographic analysis was conducted non‐invasively on rats under the anaesthetic effects of 2.5% isoflurane gas. M‐mode images were captured to assess the thickness of the interventricular septum (IVST), the posterior wall of the left ventricle (LVPWT), as well as the left ventricular internal diameter (LVD) during both diastole and systole. Subsequently, the ratio of heart mass to body weight was calculated to evaluate the presence of cardiac hypertrophy.

### 2.9 Western blotting

The extraction of proteins from animal myocardial tissue was facilitated by using RIPA buffer (P0013C; Beyotime Biotechnology, Shanghai, China). An equal amount of protein was loaded and separated via sodium dodecyl sulfate‐polyacrylamide gel electrophoresis (10% separation gel), followed by the transfer of proteins onto a PVDF membrane. After blocking the membrane with 5% skim milk for 1 h at room temperature, it was incubated with primary antibodies specific for RASD1 (1:1000), NAMPT (1:1000), p62/SQSTM1 (1:1000), LC3A/B (1:1000) and GAPDH (1:2000) overnight at 4°C. Subsequently, the membrane was washed with TBST and incubated with an HRP‐conjugated secondary antibody for 1 h at room temperature. The signal on the membrane was activated using a chemiluminescence (ECL) reagent and detected via an ECL system. Quantification of RASD1, NAMPT, and P62 protein expressions, along with the measurement of the LC3I/II ratio by Western blot, was performed using ImageJ software. The protein levels were normalized to GAPDH for standardization.

### 2.10 Real‐time quantitative PCR

Real‐time quantitative PCR was conducted to detect the mRNA levels of genes.^22^ Total RNA was isolated from heart tissue utilizing a purified SteadyPure Universal RNA Extraction Kit (AG21022, Accurate Biology, China). Subsequently, the RNA was reverse‐transcribed into cDNA using an Evo M‐MLV RT–PCR Kit (AG11728, Accurate Biology, China). Quantitative polymerase chain reaction (PCR) was performed using SYBR Green reagent kits (AG11701, Accurate Biology, China) under the following conditions: initial denaturation at 95°C for 30 s, followed by 40 cycles of denaturation at 95°C for 5 s and annealing at 60°C for 30 s. The primers employed were obtained from Accurate Biology (Hunan, China) and are detailed in Table S1.

### 2.11 Histologic and morphometric features

After sacrificing the rats, hearts were collected from each group (*n* = 6) and preserved using 4% paraformaldehyde. Subsequently, the samples underwent alcohol dehydration and were embedded in paraffin. Following paraffin embedding, the cardiac tissues were sliced into sections approximately 5 μm thick, which were then stained with haematoxylin–eosin (H&E) and wheat germ agglutinin (WGA) to assess cardiac hypertrophy.

### 2.12 Immnofluorescence staining

Immunofluorescence staining was conducted to assess the protein expression levels. Briefly, the cross‐sections were permeabilized with 0.1% Triton X‐100, followed by incubation with primary antibodies specific to RASD1 and NAMPT. To visualize the cell nuclei, DAPI dye was employed.

### 2.13 Statistical analyses

All the data have been presented as means with standard error of the mean (SEMs), analysed by GraphPad Prism 9.0 software. The normality of the data distribution was assessed using the Shapiro–Wilk test, and a *p*‐value exceeding 0.05 means closely to a normal distribution. To detect statistically significant differences among groups, we employed two‐tailed Student's *t*‐tests for comparing two groups, and one‐way ANOVA followed by Tukey's post hoc test for comparing data from more than two groups. Alternatively, the Mann–Whitney test was utilized, and *p* < 0.05 is statistically significant.

## 3 RESULTS

### 3.1 GO and KEGG pathway enrichment analysis in female‐related HCM

**FIGURE 1:**
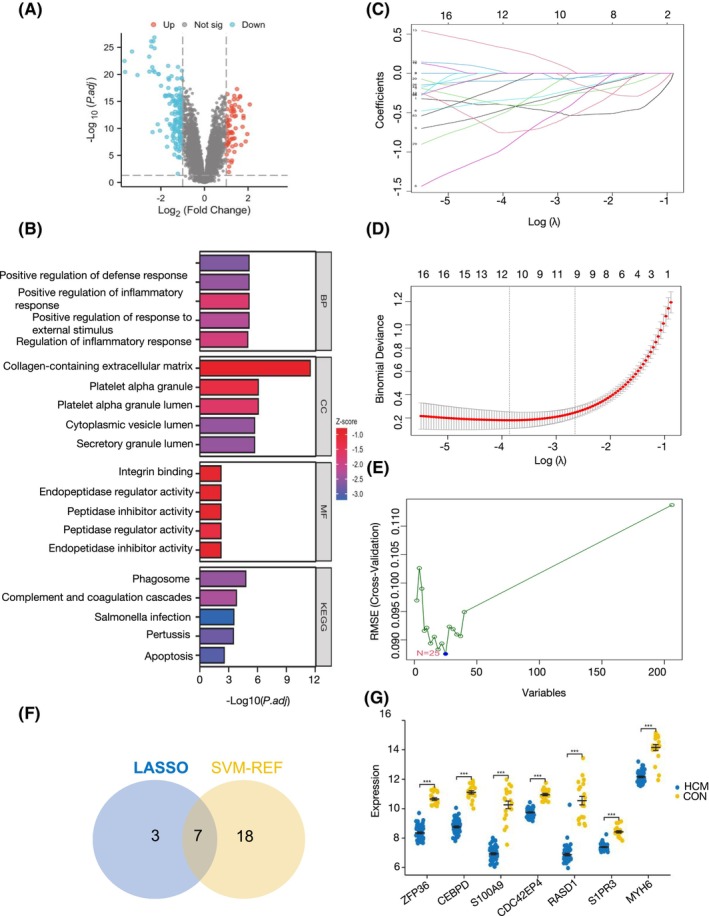
Screening of DEGs (differential expressed genes) and core genes for female‐related hypertrophic cardiomyopathy (HCM). (A) The volcano map of DEGs. (B) GO analysis and KEGG analysis of DEGs. (C) LASSO coefficient profiles of DEGs. (D) Five‐fold cross‐validation to select the optimal tuning parameter (lambda). (E) The corresponding genes screened by SVM‐RFE algorithm. (F) The venn diagram revealed the overlapping seven genes as core genes of HCM in females. (G) The scatter diagram identified the expression levels of HCM females and controlled females indicating a greatly decrease of these seven core genes (ZFP36, CEBPD, S100A9, CDC42EP4, RASD1, S1PR3 and MYH6. ***p < 0.001).

We identified 208 DEGs between the HCM females and the healthy females (Figure 1A). GO and KEGG pathway enrichment analyses elucidated the biological functions of these DEGs in HCM females (Figure 1B). Notably, the BP terms were predominantly enriched in response to lipopolysaccharide, positive regulation of defence and inflammatory responses and regulation of inflammatory response. Among the cellular component (CC) terms, the genes were particularly enriched in collagen‐rich extracellular matrix, platelet alpha granule and cytoplasmic vesicle lumen. In the molecular function (MF) category, the genes were primarily associated with integrin binding, endopeptidase regulation and peptidase inhibition. The KEGG pathway analysis revealed that these genes are primarily involved in phagosome formation, complement coagulation cascades, Salmonella infection, pertussis and apoptosis.

Based on the LASSO coefficient profiles and optimal tuning parameter selection, 10 featured genes were identified (Figure 1C,D). Additionally, a subset of 25 genes was selected using the SVM‐RFE algorithm (Figure 1E). The Venn diagram identified the overlapped 7 core genes from LASSO regression and the SVM‐RFE algorithm, including ZFP36, CEBPD, S100A9, CDC42EP4, RASD1, S1PR3 and MYH6 (Figure 1F). Notably, the expression levels of these core genes were significantly downregulated in the cardiac tissues of female HCM patients (Figure 1G).

### 3.2 Functional enrichment analysis of core genes in female‐related HCM

**FIGURE 2:**
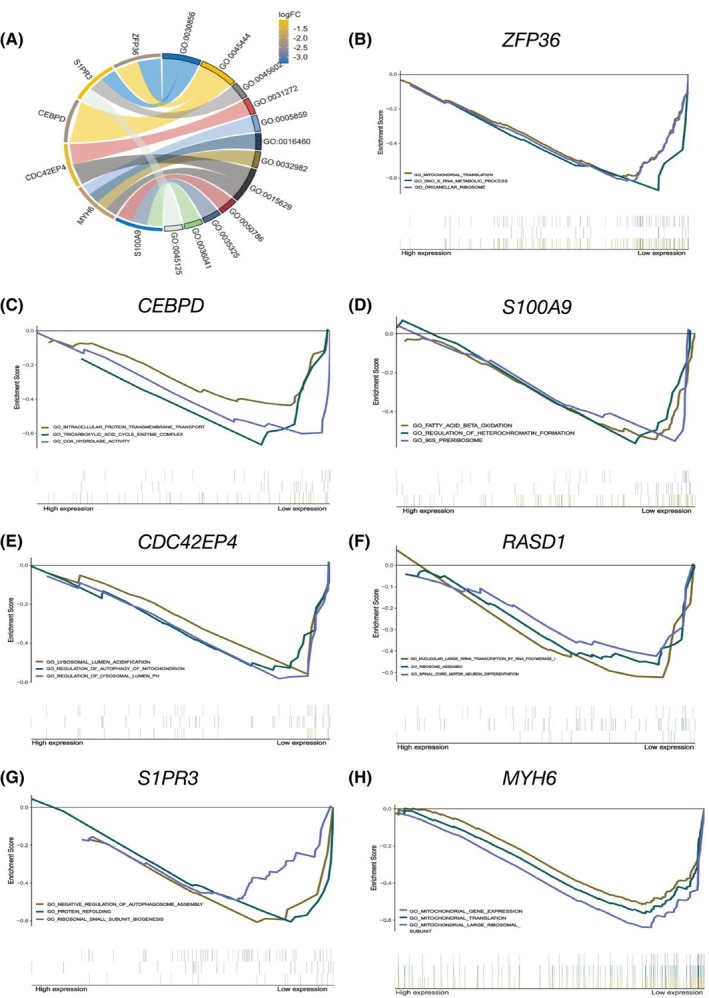
The enriched GO terms and GSEA (gene set enrichment analysis) of core genes. (A) The Chordal graph of GO terms. (B–H) GSEA revealed the enrichment in female population with different expressions of seven core gens (compared high expressions to low expressions), as ZFP36 (B), CEBPD (C), S100A9 (D), CDC42EP4 (E), RASD1 (F), S1PR3 (G) and MYH6 (H).

To gain the pathways these core genes are enriched in, we conducted GO terms to reveal mechanisms of female‐related HCM. The GO terms indicated that in the BP category, the genes were enriched in the regulation of epithelial cell differentiation, fat cell differentiation, negative regulation of endothelial cell differentiation and regulation of pseudopodium assembly. Additionally, the CC were linked to the muscle myosin complex, myosin II complex, myosin filament and actin cytoskeleton. Furthermore, the genes in the MF category were enriched in RAGE receptor binding, Toll‐like receptor, long‐chain fatty acid binding, and bioactive lipid receptor activity (Figure 2A and Table S2).

The GSEA analysis revealed enriched signalling pathways among females with high versus low expressions of seven core genes. The findings indicated that genes associated with low expression were enriched in mitochondrial translation, RNA metabolic processes, and organellar ribosome (ZFP36, Figure 2B); Intracellular protein transmembrane transport, tricarboxylic acid cycle complex, and COA hydrolase activityfatty acid β‐oxidation (CEBPD, Figure 2C); Fatty acid β‐oxidation, regulation of heterochromatin formation and 90S preribosome (S100A9, Figure 2D); Lysosomal lumen acidification, regulation of mitochondrial autophagy and lysosomal lumen (CDC42EP4, Figure 2E); Nucleolar large rRNA transcription by RNA polymerase, ribosome assembly and spinal cord motor neuron differentiation (RASD1, Figure 2F); Negative regulation of autophagosome assembly, protein refolding; ribosomal small subunit biogenesis (S1PR3, Figure 2G); Mitochondrial gene expression, translation and the mitochondrial large ribosomal subunit (MYH6, Figure 2H). The details of GSEA work were shown in Table S3.

### 3.3 Identification and validation sex differences in core genes associated with HCM and autophagy‐related signature

**FIGURE 3:**
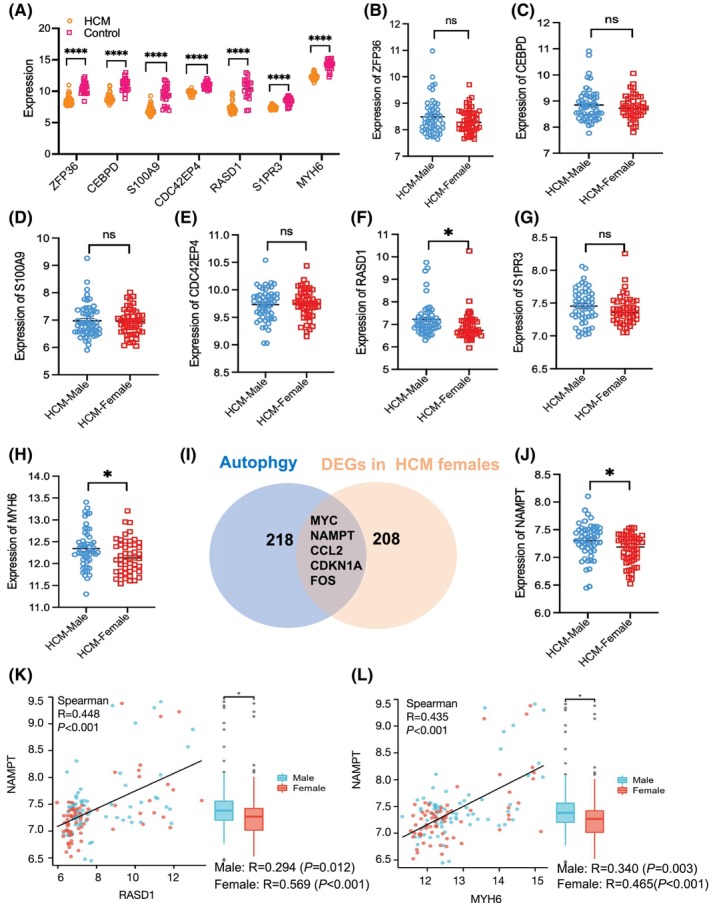
Verification the sex differences in expressions of seven core genes in hypertrophic cardiomyopathy (HCM) population, and the corerelation analysis of core genes and autophagy‐related signature. (A) The expressions of seven core genes in HCM males and control groups. (B–H) The Comparison of seven core genes levels in HCM males with those in HCM females, including ZFP36 (B), CEBPD (C), S100A9 (D), CDC42EP4 (E), RASD1 (F), S1PR3 (G) and MYH6 (H), showing significant difference expressions in RASD1 and MYH6. (*p < 0.05,****p < 0.0001, ns, not significant). (I) The venn diagram revealed the shared five genes by DEGs in female‐related genes and autophagy‐related genes from Human Autophagy Database (HADb, http://www.autophagylu/), including myc, nampt, ccl2, cdkn1a, fos. (J) The expression of NAMPT was found significant lower in tissues from HCM females than HCM males. (K) The spearman's analysis between RASD1 and NAMPT in all, female and male populations respectively. (L) The spearman's analysis between MYH6 and NAMPTin all, female and male populations respectively. Data are expressed as mean (SEM), and data were analysed by Student *t*‐test. The *p* < 0.05 was considered significant.

After analysing the data, we discovered that the expression of seven core genes was downregulated in female HCM patients compared to female controls. Additionally, we confirmed that the expression levels of these seven genes were also reduced in male HCM patients compared to male controls (Figure 3A). To further investigate whether sex differences in HCM clinical manifestations are associated with the expression levels of these seven core genes, we compared the expression levels of these genes between female and male HCM patients (Figure 3B–H). Notably, the expression levels of RASD1 (Figure 3F) and MYH6 (Figure 3H) in the heart tissue of female HCM patients were significantly lower than those in male HCM patients.

The autophagy pathway was enriched by KEGG enrichment of DEGs to female HCM patients. We further analysed the expression patterns and sex differences of autophagy‐related genes in HCM. Among the shared genes between DEGs from HCM females and autophagy‐related genes from the HADb cohort, five genes were identified, including MYC, NAMPT, C‐C motif chemokine ligand 2 (CCL2), cyclin‐dependent kinase inhibitor 1 (CKDN1A) and FOS (Figure 3I). It also revealed decreased expression levels of these five genes in male HCM patients than male controls (Table S4). When exploring sex differences in gene expression among HCM patients, we found that only NAMPT expression was significantly lower in HCM females than males (Figure 3J). It suggested a potential association between NAMPT and sex differences in HCM.

Furthermore, we conducted the correlation analysis between NAMPT and two core genes related to female‐related HCM, showing a positive correlation between NAMPT and RASD1 (*R* = 0.448, *p* < 0.001) (Figure 3K), in addition to NAMPT and MYH6 (*R* = 0.435, *p* < 0.001) (Figure 3L). Notably, these correlations were more pronounced among women, with a relatively obvious correlation between NAMPT and RASD1 in female heart tissue (*R* = 0.569, *p* < 0.001). To further validate these findings, we utilized the GSE32453 dataset (corresponding to GPL14644 platform) to explore expression patterns (Figure S1).

### 3.4 Experimental validation of sex differences in RASD1 and NAMPT expressions using animal models

**FIGURE 4:**
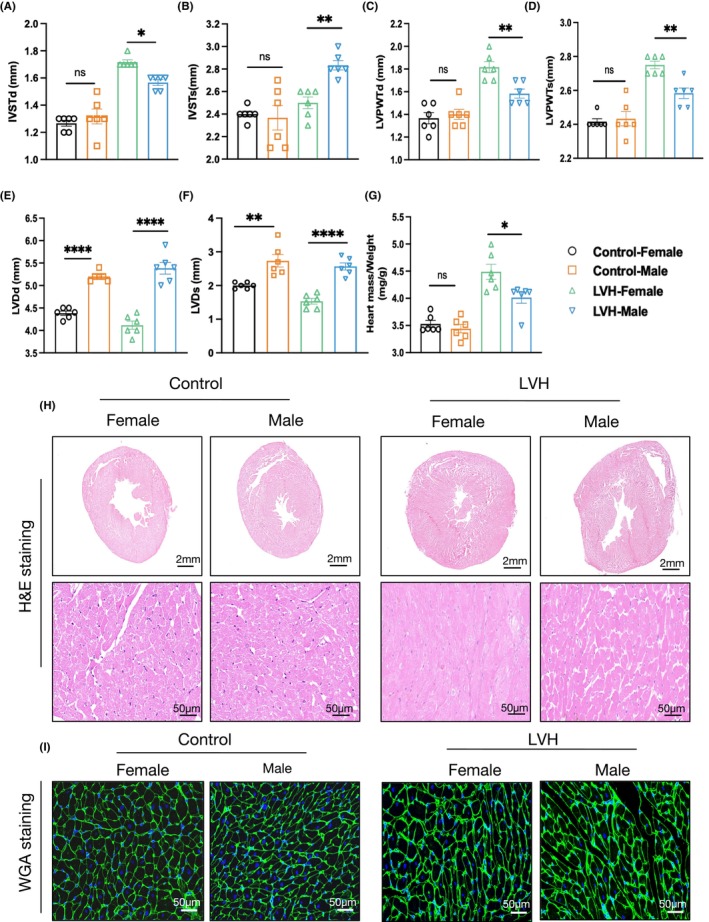
There existed sex differences in hypertrophic hearts from spontaneously hypertensive rats (SHR). (A) The thickness of interventricular septum at diastole. (B) The thickness of interventricular septum at systole. (C) The thickness of posterior wall of the left ventricle at diastole. (D) The thickness of posterior wall of the left ventricle at systole. (E) The left ventricular internal diameter at diastole. (F) The left ventricular internal diameter at systole. (G) The ratio of heart mass and weight was detected. Data are expressed as mean (SEM), and One‐way ANOVA followed by Tukey post‐hoc tests, and ajusted *p* < 0.05 was significant (*p < 0.05, **p < 0.01, ****p < 0.0001, ns, not significant). (G, H) Size of myocardial cells were assessed by staining with haematoxylin–eosin (H&E, H) (scale bar, 2 mm and 50 μm) and WGA (wheat germ agglutinin, I) (scale bar, 50 μm) in sections of hearts.

The hearts of SHR rats developed LVH by approximately 20 weeks of age, as reported.^23^ To investigate sex differences in RASD1 and NAMPT expressions, we selected SHR rats of both sexes at 6 months of age and harvested hearts, along with WKY rats as control groups. Our findings indicated that significant increases were observed in IVSTd (*p* < 0.05), IVSTs (*p* < 0.01), LVPWTd (*p* < 0.01) and LVPWTs (*p* < 0.01) of female SHR hearts compared to male SHR rats (Figure 4A–D). However, sex differences in control groups were not statistically significant. Meanwhile, measurements of the LVDs revealed significantly reduced diameters in SHR female rats compared to male rats (Figure 4E,F). Analysis of the heart mass/weight ratio (Figure 4G), H&E staining (Figure 4H), and WGA staining (Figure 4I) indicated that cardiomyocytes in the LVH‐female group, with a background of spontaneous hypertension, were significantly larger than those in the LVH‐male group.

**FIGURE 5:**
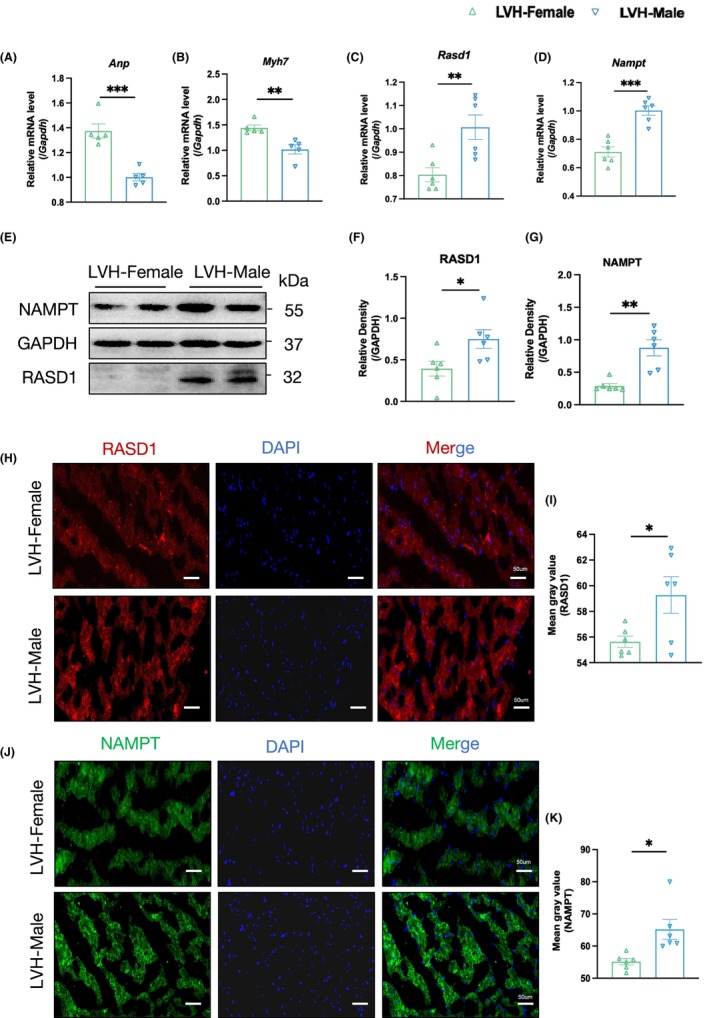
Experimental verification of sex differences in RASD1/NAMPT expressions in hearts with left ventricular hypertrophy (LVH) from spontaneously hypertensive rats (SHR). (A, B) Real‐time qPCR analysis showed the mRNA expression of Anp (A) and *Myh7* (B) in hearts with LVH from SHR were significantly higher in females than males. (C, D) Real‐time qPCR analysis of the mRNA expression of *Rasd1* (C) and *Nampt* (D) showed a reduction in SHR females than males. (E) Representative western blotting results of RASD1 and NAMPT at protein levels. (F, G) Densitometric quantification showed a significantly lower expression of NAMPT (F) and RASD1 (G) in SHR females than males (*n* = 6 per group). (H) Representative images of RASD1 expression by immunofluorescence staining of SHR hearts from females or males, and constaining with DAPI (scale bar, 50 μm) and mean grey value was presented (I). (J) Representative images of NAMPT expression by immunofluorescence staining of SHR hearts from females or males, and constaining with DAPI (scale bar, 50 μm), and mean grey value was presented (K). To identify meaningful group differences, we used two‐tailed Student's *t*‐test for comparing two groups. Otherwise, Mann–Whitney test was applied. Data are expressed as mean (SEM). For the analysis of in‐vivo experiments, the sample sizes of were at six. The mRNA and protein levels were standardized by GAPDH. (*p < 0.05, **p < 0.01, ***p < 0.001).

RT‐PCR analysis revealed significantly higher expression levels of *Anp* and *Myh7* in SHR female animals compared to males (Figure 5A,B). These findings reflect the sex differences in LVH among SHRs. Additionally, RT–PCR demonstrated marked downregulation of *Rasd1* (Figure 5C) and *Nampt* (Figure 5D) at mRNA levels in SHR females compared with males, associated with significantly reduced expressions of RASD1 and NAMPT at protein levels (Figure 5E,G). Immunofluorescence staining also identified lower RASD1 (*p* = 0.034) (Figure 5H,I) and NAMPT (*p* = 0.011) (Figure 5J,K) expressions in SHR hypertrophic hearts. Animal experiments confirmed reduced RASD1 and NAMPT expression in hypertrophic hearts from female SHRs with spontaneous hypertension.

**FIGURE 6:**
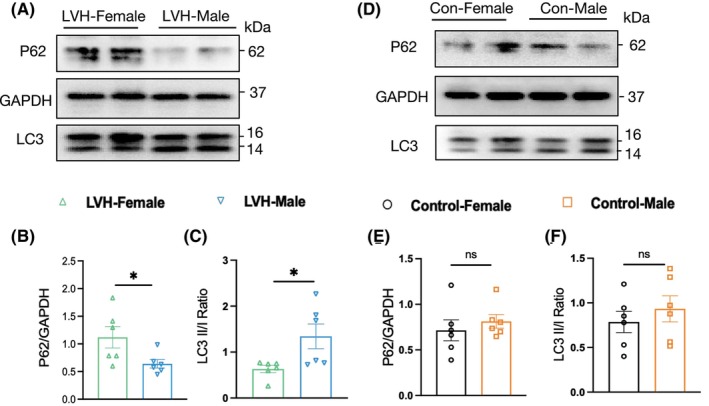
Autophagy in hearts from anmial models. (A–C) The P62 protein (A, B) expressions measured by Western blot in hypertrophic hearts from SHR females and males, in addition to a measurement of transition from LC3I to LC3II (A, C). (D–F) The P62 protein (D, E) expressions measured by Western blot in hypertrophic hearts from SHR females and males, in addition to a measurement of transition from LC3I to LC3II (D, F). Data are expressed as mean (SEM). For the analysis of in‐vivo experiments, the sample sizes of were at six, the protein levels were standardized by GAPDH. (*p< 0.05, ns, not significant).

Meanwhile, autophagic flux was examined in SHR hearts. Western blot analysis indicated increased P62 expression and decreased LC3 II/I ratio in LVH‐female group compared to LVH‐male group, indicating an inhibition of autophagy (Figure 6A–C). However, no significant sexual differences in autophagy were observed in hearts from WKY rats (Figure 6D–F).

## 4 DISCUSSION

HCM is typically characterized by asymmetric ventricular hypertrophy, affecting various anatomic regions of the heart.^24^ Numerous studies have confirmed the association of sex differences with clinical phenotypes and the prognosis of HCM, indicating that women with HCM often experience more severe cardiac dysfunction and cardiovascular risks.^13^, ^25^, ^26^ In 2021, Butters et al.^13^ described the influences of genetics and environment on the prognosis of HCM females and males. Nevertheless, there is a paucity of research examining the molecular mechanisms underlying these sex differences of HCM. It is therefore imperative to gain insight into the mechanisms and identify potential biomarkers causing HCM females suffering from higher risk of cardiovascular events and worse prognosis.^27^ Therefore, this study was conducted comparing the gene expression patterns of HCM females and males by bioinformatics, and subsequently validated using external datasets and animal models of LVH.

GSE36961, containing large HCM samples, is widely utilized for bioinformatics analyses of HCM.^28^, ^29^ However, gene expression patterns between HCM females and males were less studied. we identified 208 DEGs in female‐related HCM, enriched in inflammatory response regulation, extracellular collagen mechanisms, endopeptidase regulatory activity, cytophagocytosis and apoptosis. Additionally, using LASSO regression and SVM‐RFE algorithms, we pinpointed seven core genes (S100A9, CEBPD, S1PR3, CDC42EP4, ZFP36, RASD1 and MYH6) as being significantly involved in.

We observed a significant decrease of the expression levels of seven core genes in HCM males. Interestingly, the sex differences of these genes between HCM females and males indicated that only RASD1 and MYH6 expression levels were notably lower in female HCM patients. Consequently, we hypothesize that the downregulation of RASD1 and MYH6 expression may be associated with sexual dimorphism in HCM. Moreover, the KEGG pathway enrichment analysis of HCM females suggested a potential involvement of autophagy in its mechanisms. Among the autophagy‐related genes, we identified five genes (MYC, NAMPT, CCL2, CKDN1A and FOS) that were significantly downregulated in female HCM patients. Notably, these genes also exhibited decreased expression in male HCM patients compared to healthy controls. However, when considering sexual differences, NAMPT stood out as the gene with the most significant difference between female and male HCM patients. Furthermore, Spearman analysis revealed a stronger correlation between NAMPT and RASD1 compared to NAMPT and MYH6, particularly in female patients. The decreased expression of RASD1 and NAMPT may play a crucial role in sexual dimorphism in HCM, as supported by findings from GSE 32543 and animal studies. Additionally, more pronounced cardiac hypertrophy was observed in SHR females, characterized by reduced left ventricular inner diameters, suggesting that RASD1/NAMPT axis is prabably a key mechanism.

RASD1, formally known as Ras dexamethasone‐induced protein 1 (or AGS1), encodes for the dexamethasone‐induced Ras‐related protein 1 (Dexras1), playing a pivotal role in neuronal signal transduction.^30^ Accumulating evidence indicates a correlation between decreased RASD1 expression and the development of cardiac hypertrophy. Recent gene correlation studies in HCM patients have identified a downregulation of RASD1 expression, emphasizing its significance in HCM pathogenesis. RASD1 expression in the heart regulates cardiac ANP and BNP levels, serving as prognostic markers in failing hearts.^31^, ^32^, ^33^ Additionally, RASD1 has been identified as a novel physiologic nitric oxide effector, facilitating NO signalling through the anchoring of nNOS.^34^ This suggests that significant reductions in RASD1 levels in cardiac tissue may lead to impairments in cardiac natriuretic hormone and vasodilator functions. Furthermore, our study's single‐gene GSEA enrichment analysis revealed that low RASD1 expression is associated with nucleolar large RNA transcription by RNA polymerase, ribosome assembly, and spinal cord motor neuron differentiation. These alterations likely exacerbate cardiac hypertrophy and remodelling in HCM patients.

Furthermore, our findings also indicate a positive correlation between RASD1 and NAMPT expression. RASD1 has been shown to influence the AKT/mTOR signalling pathway, which regulates NAMPT activity in cardiomyocytes. NAMPT, a crucial enzyme in the nicotinamide adenine dinucleotide (NAD+) salvage pathway, is ubiquitous in cells and tissues, playing a pivotal role in mitochondrial protein expression, oxidative metabolism, bioenergetics, and autophagy.^35^, ^36^ As previous studies have indicated that autophagy can reduce oxidative damage by removing aggregated proteins and damaged mitochondria, playing an important role in regulating diverse diseases, including cancer and cardiac hypertrophy. Moreover, the inhibition of autophagy could exacerbate cardiac hypertrophy.^37^, ^38^ Deng et al. found that Nampt could induce autophagy by targeting AMPK/mTOR signalling.^39^ A recent research has shown that NAMPT enhances antioxidant defence in diabetic cardiomyopathy through the NADPH‐dependent reduction system.^40^ Knockdown of Nampt significantly increases cardiac systolic dysfunction and cardiomyocyte apoptosis under stress conditions, while overexpressed NAMPT can reduce cardiomyocyte apoptosis. These findings suggest a protective role for NAMPT in cardiomyocytes.^41^ In current study, it have confirmed that female HCM patients exhibit significantly lower NAMPT expression in heart tissue compared to male HCM patients, along with impaired autophagy in cardiomyocytes. This abnormal cardiac energy metabolism and oxidative stress damage associated with downregulated NAMPT expression may partially explain why women with HCM are at a higher risk of cardiovascular disease compared to men. Therefore, it demonstrates that oxidative stress, energy synthesis, and disrupted autophagy balance in cardiomyocytes resulting from downregulation of the RASD1/NAMPT axis may underlie the increased cardiac hypertrophy and cardiovascular risk observed in female HCM patients. It provides new biological indicators, in addition to potential therapeutic mechanisms and targets for predicting clinical prognosis of HCM patients, which requires further clinical investigation by multi‐omics anlysis validation.^42^, ^43^

There still exists limitations in this study. Actually, bioinformatics and external validation suggest that downregulation of RASD1/NAMPT may be an important mechanism for the increased risk of cardiovascular disease in women with HCM. Although we have preliminarily validated gender differences in HCM patients with GSE32453 (corresponding to GPL14644 platform), a larger population dataset is needed for validation. Moreover, it requires a further validation to explore RASD1/NAMPT as biomarkers for predicting higher cardiovascular events in LVH disorders.

## 5 CONCLUSIONS

Therefore, the RASD1/NAMPT axis emerges as a crucial cellular signalling pathway implicated in the pathogenesis of cardiac hypertrophy. Furthermore, the expression levels of RASD1 and NAMPT could serve as potential prognostic biomarkers for patients with HCM, necessitating the implementation of cardiac surveillance, early detection and tailored therapeutic strategies for these individuals.

## AUTHOR CONTRIBUTIONS

**Hongyu Kuang:** Conceptualization (lead); data curation (lead); formal analysis (lead); methodology (equal); software (equal); validation (equal); writing – original draft (lead). **Yanping Xu:** Formal analysis (equal); methodology (equal); validation (equal). **Guangliang Liu:** Formal analysis (supporting); methodology (supporting); software (equal). **Yuhao Wu:** Formal analysis (supporting); methodology (supporting); software (equal). **Zhiyan Gong:** Data curation (supporting); validation (supporting). **Yuehui Yin:** Conceptualization (equal); supervision (lead); writing – review and editing (lead).

## FUNDING INFORMATION

This study was supported by the Chongqing Natural Science Foundation (CSTB2023NSCQ‐BHX0044).

## CONFLICT OF INTEREST STATEMENT

The authors declare that no conflict of interest exist in this study.

## Supporting information


Data S1:

## Data Availability

The open‐access datasets are available through the following URL: https://www.ncbi.nlm.nih.gov database annotated by GPL10558 as a Series Matrix File.
