# Supplementary material for: Integration analysis using bioinformatics and experimental validation on cellular signalling for sex differences of hypertrophic cardiomyopathy
Source: J Cell Mol Med. 2024 Nov 13;28(21):e70147. doi: 10.1111/jcmm.70147 (PMC11558267; doi:10.1111/jcmm.70147)
Supplement: Supplementary file 1 — Data S1: [file JCMM-28-e70147-s001.docx]

***Supplemental Material***

**Integration analysis using bioinformatics and experimental validation on cellular signaling for sex differences of hypertrophic cardiomyopathy**

4 Tables and 1 Figrues.

**Table S1**

**Table S1 Primer sequences for qPCR**

| **Gene Name** | **Specie** | **Primers (5` - 3`)** |
| --- | --- | --- |
| *Anp* | Rat | F: CTGAGAGGTGGTGAATACCCT  R: GTCCGTGGTGCTGAAGTTTA |
| *Bnp* | Rat | F: GCTCTCAAAGGACCAAGGC  R: GCTCTCAAAGGACCAAGGC |
| *Myh7* | Rat | F: AAGAGCCGTGACATTGGC  R: TTGCTTTATTGTGTTTCTGCCT |
| *Rasd1* | Rat | F: TTCTCTCAGCCACGCATCTG  R: CCGTCTTGCCCACTTTGGAT |
| *Nampt* | Rat | F: GTTGCTGCCACCTTACCTTAGAG  R: CCACCAGAACCAAAGGAGACATT |
| *Gapdh* | Rat | F: GGCACAGTCAAGGCTGAGAATG  R: ATGGTGGTGAAGACGCCAGTA |

**Table S2**

**Table S2 The detailed description of GO Terms**

| **Ontology** | **ID** | **Description** | **GeneRatio** | **BgRatio** | ***P*-value** |
| --- | --- | --- | --- | --- | --- |
| BP | GO:0030856 | regulation of epithelial cell differentiation | 2/7 | 157/18800 | 0.0014 |
| BP | GO:0045444 | fat cell differentiation | 2/7 | 237/18800 | 0.0032 |
| BP | GO:0045602 | negative regulation of endothelial cell differentiation | 1/7 | 12/18800 | 0.0045 |
| BP | GO:0031272 | regulation of pseudopodium assembly | 1/7 | 13/18800 | 0.0048 |
| CC | GO:0005859 | muscle myosin complex | 1/7 | 15/19594 | 0.0053 |
| CC | GO:0016460 | myosin II complex | 1/7 | 22/19594 | 0.0078 |
| CC | GO:0032982 | myosin filament | 1/7 | 22/19594 | 0.0078 |
| CC | GO:0015629 | actin cytoskeleton | 2/7 | 499/19594 | 0.0125 |
| MF | GO:0050786 | RAGE receptor binding | 1/7 | 10/18410 | 0.0038 |
| MF | GO:0035325 | Toll-like receptor binding | 1/7 | 12/18410 | 0.0046 |
| MF | GO:0036041 | long-chain fatty acid binding | 1/7 | 15/18410 | 0.0057 |
| MF | GO:0045125 | bioactive lipid receptor activity | 1/7 | 16/18410 | 0.0061 |

**Table S3**

**Table S3 Statistical results from GSEA work**

| Gene | GO-BP | GO-CC | GO-MF |
| --- | --- | --- | --- |
| ZFP36 | Mitochondrial translation  (NES=-1.86, P=0.002) | Organellar ribosome  (NES=-1.90, P<0.001) | SNO-S-RNA-metabolic process  (NES=-1.89, P<0.001) |
| CEBPD | Intracellular protein transmembrane transport (NES=-1.77, P=0.002) | COA Hydrolase activity  (NES=-1.85, P=0.002) | Tricarboxylic acid cycle enzyme complex  (NES=-1.66, P=0.018) |
| S100A9 | Fatty acid beta oxidation  (NES=-1.80, P<0.001) | 90S-preribosome  (NES=-1.81, P=0.002) | Regulation of heterochromatin formation (NES=-1.80, P=0.004) |
| CDC42EP4 | Lysosomal lumen acidification (NES=-1.78, P<0.001) | Regulation of lysosomal lumen PH (NES=-1.77, P<0.001) | Regulation of autophagy of mitochondrion (NES=-1.77, P=0.006) |
| RASD1 | Nucleolar large RRNA transcription by RNA Polymerase I (NES=-1.84, P=0.004) | Spinal cord MoTOR neuron differentiation (NES=-1.59,P=0.008) | Ribosome Assembly  (NES=-1.68, P=0.004) |
| S1PR3 | Negative regulation of autophagosome assembly  (NES=-1.66, P=0.016) | Ribosomal small subunit biogenesis  (NES= -1.81, P<0.00`) | Protein refolding  (NES=-1.77, P=0.002) |
| MYH6 | Mitochondrial gene expression (NES=-1.70, P=0.023) | Mitochondrial large ribosomal subunit(NES=-1.75, P=0.002) | Mitochondrial translation (NES=-1.74, P=0.014) |

**Table S4**

**Table S4 The differences of expressions in 5 autophagy-related genes**

| **Gene Name** | **HCM vs Control (in Males)** | **HCM-Female vs HCM-Male** |
| --- | --- | --- |
| *MYC* | *p*<0.0001 | *p*=0.8579 |
| *NAMPT* | *p*<0.0001 | *p*=0.0044 |
| *CCL2* | *p*<0.0001 | *p*=0.6196 |
| *CDKN1A* | *p*<0.0001 | *p*=0.1031 |
| *FOS* | *p*<0.0001 | *p*=0.7731 |

**Fig.S1**

**
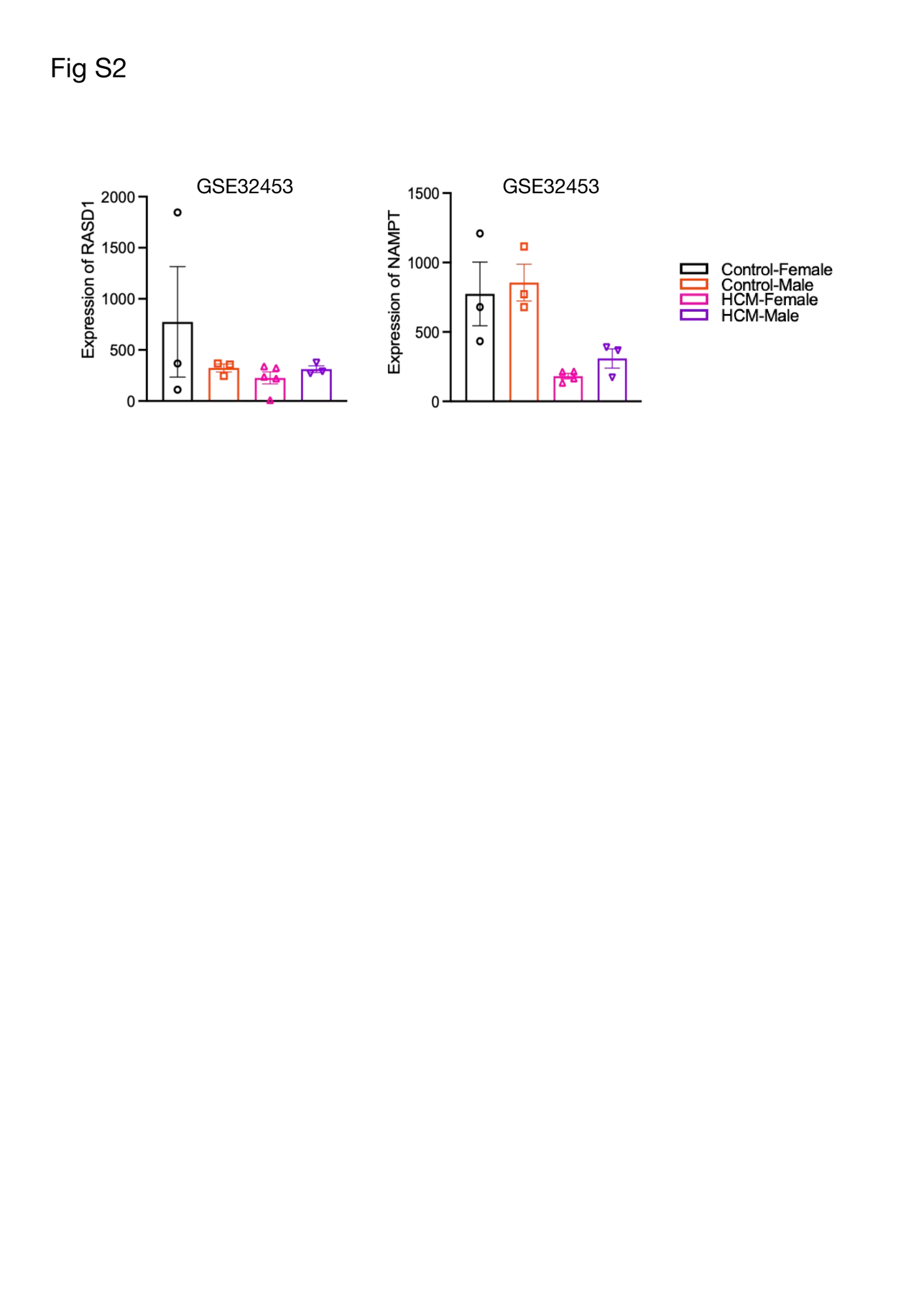
**

Fig.S1 The GSE32453 dataset (corresponding to GPL14644 platform) was ultilized to validate expression patterns.
